# Clinical efficacy of prophylactic intravenous immunoglobulin for elderly DLBCL patients with hypogammaglobulinemia in the COVID-19 pandemic era

**DOI:** 10.3389/fonc.2024.1380492

**Published:** 2024-04-23

**Authors:** Dong Won Baek, Ga-Young Song, Ho Sup Lee, Young Rok Do, Ji Hyun Lee, Ho-Young Yhim, Joon Ho Moon, Deok-Hwan Yang

**Affiliations:** ^1^ Department of Hematology, Kyungpook National University Hospital, School of Medicine, Kyungpook National University, Daegu, Republic of Korea; ^2^ Department of Hematology, Chonnam National University Hwasun Hospital, Chollanamdo, Republic of Korea; ^3^ Division of Hematology, Department of Internal Medicine, Kosin University College of Medicine, Kosin University Gospel Hospital, Busan, Republic of Korea; ^4^ Department of Hematology, Dongsan Medical Center, Keimyung University School of Medicine, Daegu, Republic of Korea; ^5^ Division of Hematology, Department of Internal Medicine, Dong-A University College of Medicine, Busan, Republic of Korea; ^6^ Department of Internal Medicine, Jeonbuk National University Medical School, Jeonju, Republic of Korea

**Keywords:** COVID-19, diffuse large B-cell lymphoma, elderly, hypogammaglobulinemia, immunochemotherapy

## Abstract

**Background:**

Elderly patients diagnosed with diffuse large B-cell lymphoma (DLBCL) undergoing reduced intensity R-CHOP therapy are at a heightened risk of acquiring infections, notably coronavirus disease 2019 (COVID-19) infection. This study aimed to evaluate the efficacy of intravenous immunoglobulin (IVIG) as prophylaxis against COVID-19 in this vulnerable population.

**Methods:**

A total of 125 elderly patients with DLBCL undergoing reduced intensity R-CHOP therapy were analyzed in this prospective, multicenter study. Patients with hypogammaglobulinemia were categorized into IVIG and non-IVIG groups, while those with normal immunoglobulin levels constituted the observation group. The study evaluated COVID-19 infection rates, therapy response, and safety outcomes.

**Results:**

Among the enrolled patients (median age: 77 years), 89 patients (71.2%) presented with hypogammaglobulinemia at diagnosis, and 56 patients enrolled in the IVIG administration group. IVIG administration remarkably reduced COVID-19 infection rates compared to non-IVIG recipients (8.9% vs. 24.6%; p =0.040). Notably, patients over 80 years old were more susceptible to COVID-19. Patients on IVIG exhibited good tolerance with manageable adverse events. Among patients with hypogammaglobulinemia who received IVIG, 40.5% of patients developed additional immunoglobulin deficiencies during chemotherapy. One or more new hypogammaglobulinemia occurred during chemotherapy in 72% of patients with hypogammaglobulinemia who did not receive IVIG, and in 61.3% of patients who did not have hypogammaglobulinemia at diagnosis.

**Conclusion:**

IVIG showed promise in reducing COVID-19 infections among elderly patients with DLBCL receiving reduced intensity R-CHOP therapy. This highlights IVIG’s potential as a prophylactic measure, necessitating further investigation to optimize dosing, administration schedules, and potential interactions with vaccination strategies.

## 1 Introduction

Severe acute respiratory syndrome coronavirus 2 (SARS-CoV-2) has spread worldwide since December 2019, causing coronavirus disease 2019 (COVID-19) in numerous individuals. Notably, patients with hematologic malignancies have exhibited increased vulnerability to COVID-19 infection, leading to higher incidence rates and more adverse treatment outcomes compared to the general population (1–3). A Spanish research group reported that individuals with lymphoma over 70 years old faced a higher risk of mortality to COVID-19. Moreover, an active disease status considerably escalated the risk of death (4). Particularly, the mortality risk associated with COVID-19 in patients with diffuse large B-cell lymphoma (DLBCL) was noted to be higher than in other B-cell lymphomas such as follicular lymphoma (4).

SARS-CoV-2 continues to cause a global pandemic, and it has become evident that COVID-19’s clinical course is notably severe among patients with DLBCL (5, 6). With the introduction of vaccines targeting SARS-CoV-2, recommendations have been made for the vaccination of elderly patients with DLBCL (5). Vaccination is beneficial in preventing hospitalizations and deaths post-COVID-19 infection (7). However, most trials excluded individuals with malignancies, resulting in limited data concerning vaccine safety and efficacy for patients with DLBCL (8, 9). The immune response to SARS-CoV-2 vaccines may be significantly impaired, especially in patients undergoing B cell–depleting treatments (10). Moreover, there are still no clear standard guidelines for DLBCL patients infected with SARS-CoV-2 during immunochemotherapy (11, 12).

Meanwhile, approximately 15%–20% of patients with DLBCL display a reduction in at least one immunoglobulin class at diagnosis (13–15). Moreover, during B cell–depleting therapies such as rituximab, over 30% of patients with B-cell lymphoma newly develop some form of immunoglobulin deficiency (14). Hypogammaglobulinemia, characterized by reduced serum immunoglobulin G (IgG), immunoglobulin A (IgA), or immunoglobulin M (IgM) levels, is commonly observed in chronic lymphocytic leukemia (CLL) with an incidence ranging from 20% to 70% (16). IgM is a critical primary responder to viral pathogens causing major pandemics, and IgG responses are fundamental to adaptive immunity (17, 18). Emerging research is shedding light on the diverse functions of IgA in mucosal defense against viral infections, while the role of IgD in viral infections remains poorly elucidated (19, 20). Intravenous immunoglobulin (IVIG), primarily containing over 95% IgG and minute amounts of IgM or IgA derived from human plasma, may potentially offer clinical benefits in preventing or treating viral infections (21). However, the COVID-19 treatment guidelines currently discourage the use of IVIG in adults due to uncertainties regarding whether IVIG products contain SARS-CoV-2 neutralizing antibodies from pooled donor plasma. Moreover, clinical trials evaluating the therapeutic effect of SARS-CoV-2 hyperimmunoglobulin excluded patients with cancer (22, 23). Nonetheless, some positive findings regarding IVIG against COVID-19 infections have emerged. A British study examining anti-SARS-CoV-2-spike antibody titers before and after IVIG infusion in 35 patients with primary immunodeficiencies who regularly received IVIG demonstrated an increase in antibody titers and serum neutralization capacity post-IVIG infusion in most patients (24). However, a paucity of clinical studies exploring the effects of IVIG on the treatment or prevention of COVID-19 in patients with hematologic malignancies remains.

Previous research on CLL has highlighted the significant association between hypogammaglobulinemia and an increased risk of infectious events, a primary cause of death. Thus, patients with CLL with hypogammaglobulinemia might benefit from antibody replacement therapy using IVIG (16). Similarly, patients with hypogammaglobulinemia undergoing immunochemotherapy for DLBCL experience higher rates of severe infections, indicating potential benefits of IVIG for infection prevention (13, 25). Thus, this prospective interventional study aimed to investigate the clinical impact of prophylactic IVIG on elderly patients newly diagnosed with DLBCL receiving reduced intensity R -CHOP therapy, in the context of protection against COVID-19 infections.

## 2 Methods

### 2.1 Study design and participants

This prospective, interventional, multicenter study was conducted from June 2021 to 2023 to evaluate the efficacy of IVIG in preventing COVID-19 infection in newly diagnosed elderly patients with DLBCL undergoing immunochemotherapy. Inclusion criteria encompassed patients aged 70 years or older, recently diagnosed with DLBCL, and suitable for reduced intensity R-CHOP therapy without contraindications for IVIG administration. The Ann Arbor staging system was used for staging, and risk stratification was determined using the international prognostic index (26, 27). Patients with primary central nervous system lymphoma, secondary transformed DLBCL, and human immunodeficiency virus-associated lymphoma were excluded. Serum IgG, IgA, IgM, and IgD levels were assessed at diagnosis, with any class falling below the reference range classified as hypogammaglobulinemia. Patients with hypogammaglobulinemia had the option to participate in a hypogammaglobulinemia group and select whether to receive IVIG as part of the study. The clinical courses of patients with hypogammaglobulinemia who did not receive IVIG and those with normal immunoglobulin levels were monitored as an observation group. All patients provided informed consent following the Declaration of Helsinki guidelines. The Institutional Review Board of Kyungpook National University Hospital (KNUH 2020-03-026) and each other participating center approved this study.

### 2.2 Procedures

All enrolled patients underwent six cycles of reduced intensity R-CHOP therapy administered every 3 weeks. Treatment involved IV administration of rituximab at 375 mg/m^2^, cyclophosphamide at 600 mg/m^2^, doxorubicin at 30 mg/m^2^, vincristine at 1.0 mg on day 1, and oral prednisone at 40 mg/m^2^ on days 1–5. Pegylated granulocyte colony-stimulating factor was administered on day 2 of each chemotherapy cycle. Patients in the study group received 400 mg/kg of IVIG on days 3 and 11 during the first reduced intensity R-CHOP cycle, followed by 400 mg/kg of IVIG on day 3 from the second to the sixth cycle (**Supplementary Table S1**).

COVID-19 diagnosis was established when there is positivity for SARS-CoV-2 via reverse transcriptase-polymerase chain reaction (RT-PCR). Testing for COVID-19 was routinely performed before each chemotherapy for screening, and the test was repeated whenever there were unusual symptoms such as fever or sore throat. Serum immunoglobulin levels were assessed at interim and end-of-treatment evaluations, monitored at 3-month intervals post-treatment. Prophylactic agents for bacterial, viral, fungal, and pneumocystis pneumonia infections were permitted as per the investigator’s discretion. When patients required hospitalization and received intravenous antibiotic treatment, a severe infection was considered. Patients may withdraw from the study in cases of disease progression or upon personal request.

### 2.3 Outcomes

Patients were stratified into three groups: hypogammaglobulinemia with IVIG, hypogammaglobulinemia without IVIG, and normal immunoglobulin levels. The primary endpoint involved comparing COVID-19 infection rates during chemotherapy among these three groups. Safety assessments were conducted at each patient visit, and toxicity was evaluated as per the Common Terminology Criteria for Adverse Events version 5.0 (NIH, Bethesda, MD, USA). This clinical trial is registered as KCT0005626 in the clinical research information service (https://cris.nih.go.kr).

### 2.4 Statistical analysis

Using medians for continuous variables and frequency percentages for categorical variables, patient characteristics were summarized. Fisher’s exact test was employed for categorical variables, while the Wilcoxon rank-sum test was utilized for continuous variables in between-group comparisons. To identify risk factors for COVID-19 infection, Cox regression analysis was employed. Variables significantly associated with COVID-19 infection in univariate analysis (p ≤ 0.1) were included in the multivariate analysis. Hazard ratios and 95% confidence intervals were estimated for each factor, with statistical significance set at p < 0.05. Statistical analysis was conducted using R statistical software (version 4.3.1; R Foundation for Statistical Computing, Vienna, Austria, available at http://www.r-project.org).

## 3 Results

### 3.1 Patient characteristics

In this study, data from a total of 125 patients newly diagnosed with DLBCL were collected and analyzed. **Table 1** summarizes the patient characteristics. The median age of all enrolled patients was 77 years, and 70 patients (56.0%) were male. A total of 89 patients (71.2%) exhibited decreased levels in one or more immunoglobulin classes at diagnosis, with 56 patients enrolled in the IVIG administration group. The median age of the IVIG group was 77 years, and 16 patients (28.6%) were over 80 years old. Before diagnosis, five patients in the IVIG group and four patients in the non-IVIG group had prior COVID-19 infections. In both groups, most patients received prophylactic antibiotics and antifungal agents during chemotherapy. **Table 2** outlines the prevalence of hypogammaglobulinemia by class. In the IVIG group, decreased IgM and IgD levels were the most common, identified in 34 (60.7%) and 29 patients (51.8%), respectively. Sixteen patients (28.6%) demonstrated two or more overlapping decreased immunoglobulin levels. Thirty-three patients with hypogammaglobulinemia did not receive IVIG infusion, and in this non-IVIG group, decreased IgM and IgD levels were also prevalent, with five patients revealing two or more hypogammaglobulinemia classes.

**Table 1:** Patient characteristics.

	Hypogammaglobulinemia (n = 89)		Normal immunoglobulin level (n = 36)	p-value
	IVIG (n = 56)	Non-IVIG (n = 33)		
Age, median (range)	77 (70–90)	79 (70–85)	77.5 (70–86)	0.965
≥80, n (%)	16 (28.6)	13 (39.4)	12 (33.3%)	
Sex				0.534
Male, n (%)	30 (53.6)	16 (48.5)	24 (66.7)	
Elevated LDH, n (%)	35 (62.5)	23 (69.7)	22 (61.1%)	0.723
ECOG, n (%)				0.192
0–1	31 (55.4)	11 (33.3)	20 (55.6)	
2–3	25 (44.6)	22 (66.7)	16 (44.4)	
EN involvement, n (%)				0.053
0	26 (46.4)	6 (18.2)	13 (36.1)	
1	21 (37.5)	15 (45.5)	10 (27.8)	
≥2	9 (16.1)	12 (36.4)	13 (36.1)	
Ann Arbor stage, n (%)				0.453
1	4 (7.1)	5 (15.2)	4 (11.1)	
2	19 (33.9)	10 (30.3)	13 (36.1)	
3	13 (23.2)	3 (9.1)	9 (25.0)	
4	20 (35.7)	15 (45.5)	10 (27.8)	
IPI				0.221
1	12 (21.4)	11 (33.3)	3 (8.3)	
2	11 (19.6)	3 (9.1)	6 (16.7)	
3	11 (19.6)	8 (24.2)	15 (41.7)	
4	16 (28.6)	6 (18.2)	9 (25.0)	
5	6 (10.7)	5 (15.2)	3 (8.3)	
No. of COVID-19 vaccinations, n (%)				0.684
none	31 (55.4)	18 (54.5)	21 (58.3)	
1	16 (28.6)	8 (24.2)	9 (25.0)	
2	9 (16.1)	7 (21.2)	6 (16.7)	
*Prior COVID-19 infection, n (%)	5 (8.9)	1 (3.0)	3 (8.3)	0.555
Prophylactic antibiotics, n (%)	54 (96.4)	33 (100.0)	36 (100.0)	0.286
Prophylactic antiviral agent, n (%)	25 (44.6)	21 (63.6)	15 (41.7)	0.545
Prophylactic anti-PCP agent, n (%)	23 (41.1)	25 (75.8)	21 (58.3)	0.116
Prophylactic antifungal agent, n (%)	50 (89.3)	30 (90.9)	32 (88.9)	0.278

COVID-19, coronavirus disease 2019; ECOG, Eastern Cooperative Oncology Group; EN, extra nodal; IPI, International Prognostic Index; IVIG, intravenous immunoglobulin; LDH, lactate dehydrogenase; PCP, pneumocystis pneumonia.

*History of past COVID-19 infection before diagnosis of DLBCL.

**Table 2:** Distribution of hypogammaglobulinemia at diagnosis.

Immunoglobulin	IVIG (n = 56)	Non-IVIG (n = 33)	p-value
IgG, n (%)	9 (16.1)	3 (9.1)	0.542
IgA, n (%)	4 (7.1)	3 (9.1)	1.000
IgM, n (%)	34 (60.7)	17 (51.5)	0.701
IgD, n (%)	29 (51.8)	16 (48.5)	0.421

IgA, immunoglobulin A; IgD, immunoglobulin D; IgG, immunoglobulin G; IgM, immunoglobulin M; IVIG, intravenous immunoglobulin.

In the IVIG group, 16 patients demonstrated overlapped hypogammaglobulinemia, and in the non-IVIG group, five patients had overlapped hypogammaglobulinemia.

### 3.2 Response to therapy and outcomes

**Figure 1:**
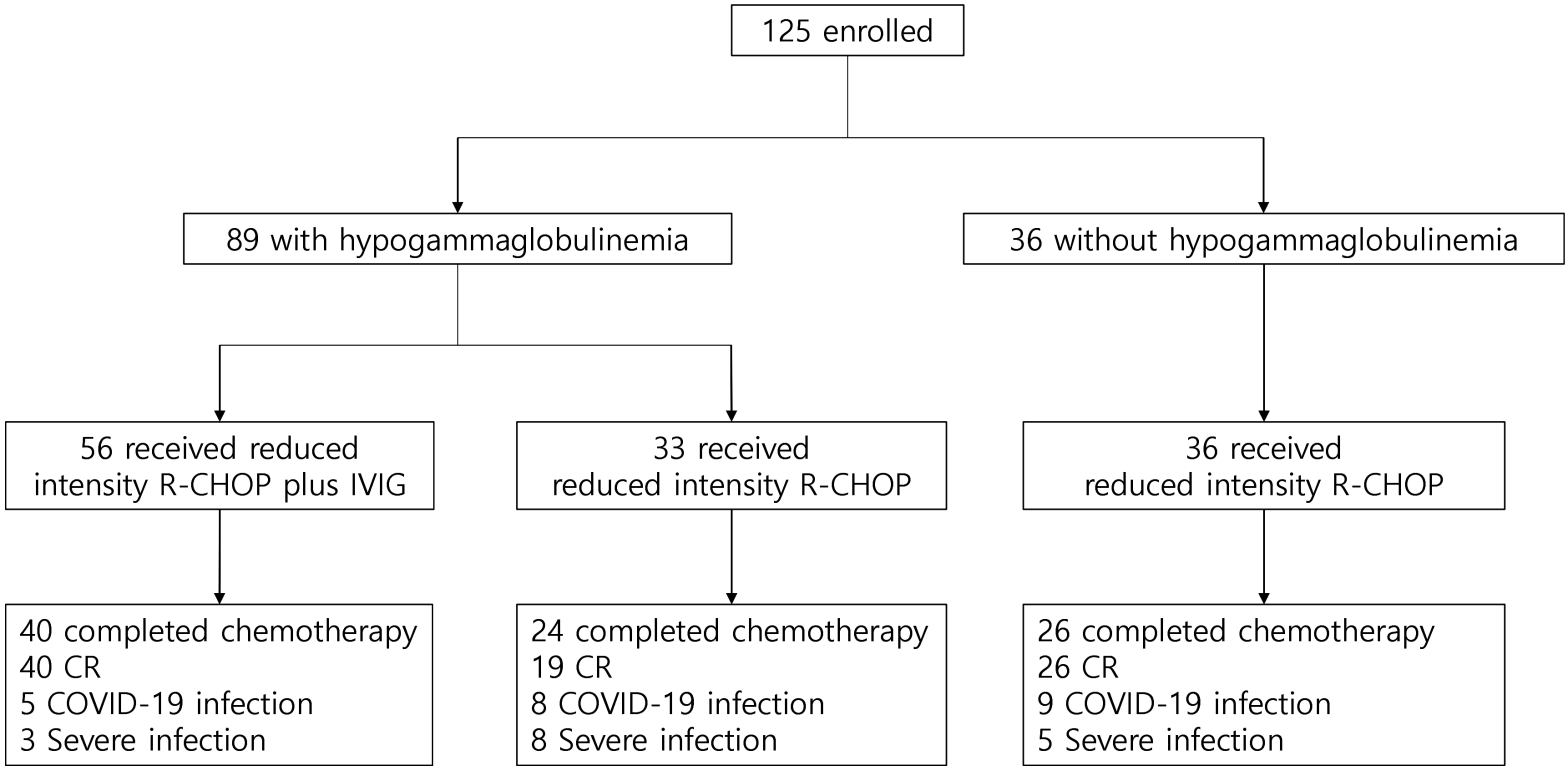
Study profile. COVID-19, coronavirus disease 2019; CR, complete response; IVIG, intravenous immunoglobulin; R-CHOP, immunochemotherapy including rituximab, cyclophosphamide, doxorubicin, vincristine, and oral prednisone.

In the IVIG administration group, 16 of 56 patients discontinued treatment. The reasons were as follows: nine patients withdrew due to severe general weakness, three patients discontinued reduced intensity R-CHOP due to disease progression, and two deaths were documented during chemotherapy (one cerebral hemorrhage associated with a fall and one liver failure from chronic hepatitis B). Furthermore, two patients could not continue immunochemotherapy due to aspiration pneumonia and cardiac issues (non-ST-elevation myocardial infarction [NSTEMI]), respectively. Eventually, 40 patients completed the scheduled reduced intensity R-CHOP and IVIG prophylaxis. A total of 40 patients achieved complete remission (CR). Adverse effects associated with IVIG included manageable symptoms such as fever, chills, nausea, and vomiting. One patient discontinued the study due to chest pain during IVIG administration (**Supplementary Table S2**). Among patients with hypogammaglobulinemia who did not receive IVIG, 24 completed six cycles of reduced intensity R-CHOP, with 19 achieving CR. Five patients discontinued treatment due to general weakness, and three had disease progression during chemotherapy. In the group with normal immunoglobulin levels at diagnosis, 26 of 36 patients achieved CR, while 10 patients discontinued treatment due to general weakness (n = 6) or disease progression (n = 4) (**Figure 1**).

### 3.3 IVIG and COVID-19 infection

**Figure 2:**
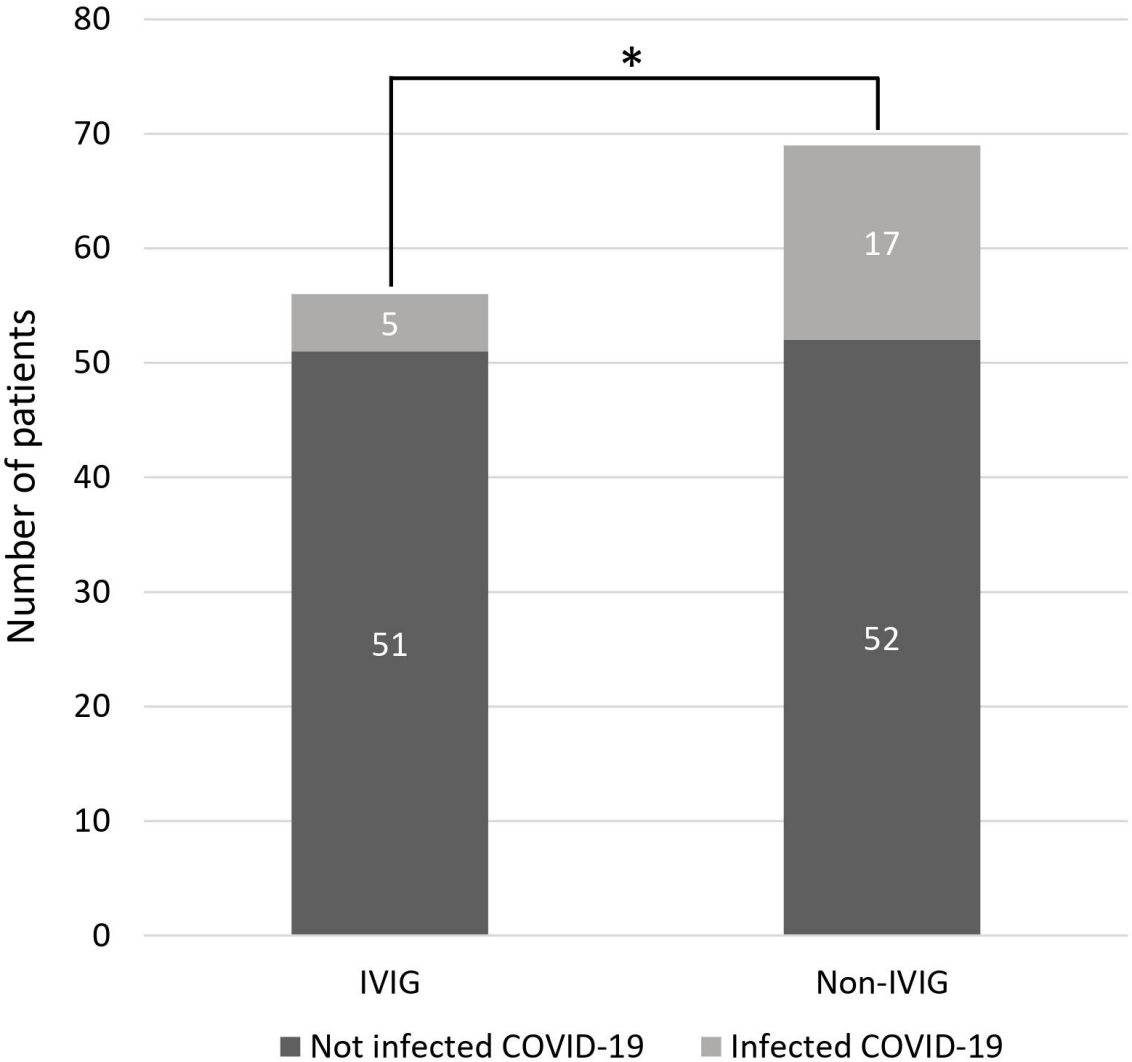
Comparison of COVID-19 infection between the IVIG and non-IVIG group (*p = 0.040). COVID-19, coronavirus disease 2019; IVIG, intravenous immunoglobulin.

**Figure 3:**
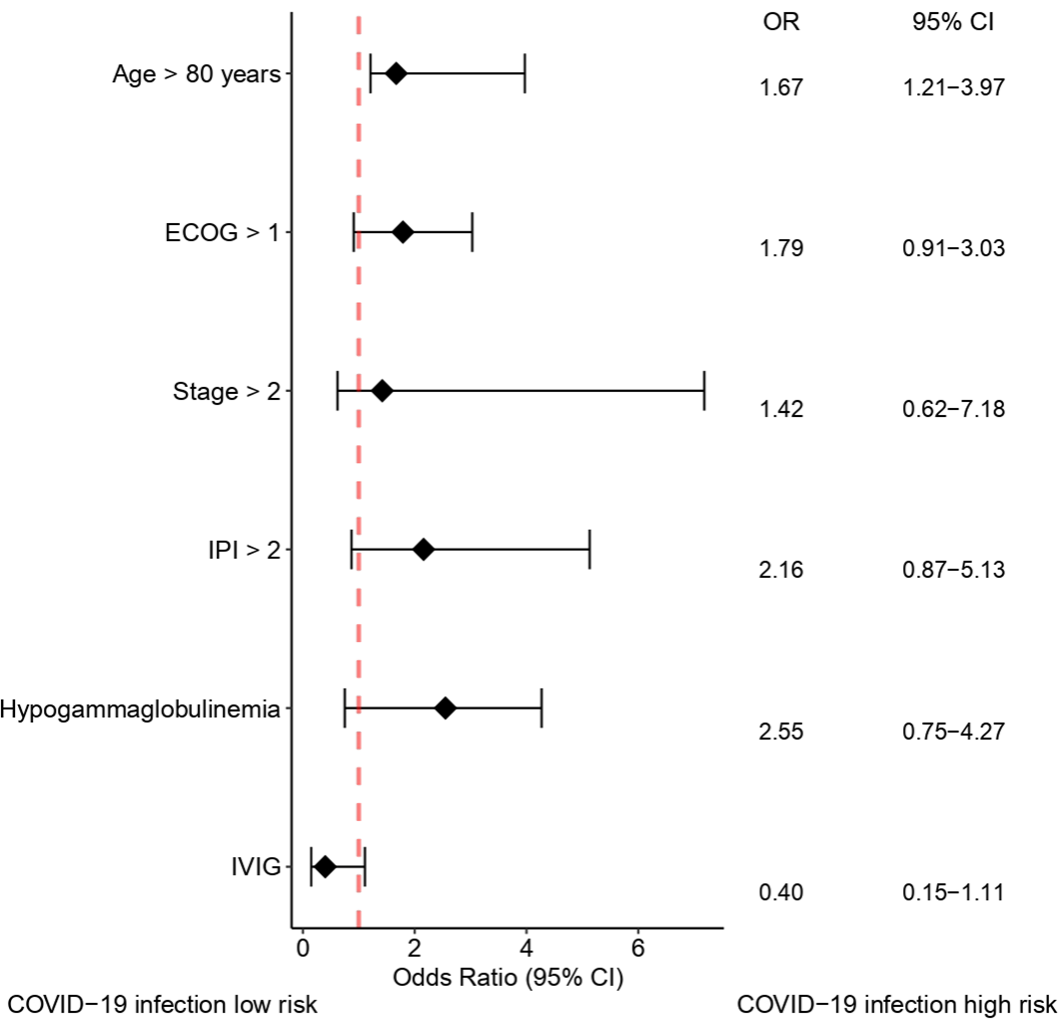
Forest plot showing factors affecting COVID-19 infection. COVID-19, coronavirus disease 2019; CI, confidence interval; ECOG, Eastern Cooperative Oncology Group; IVIG, intravenous immunoglobulin; IPI, International Prognostic Index; OR, odds ratio.

In patients with hypogammaglobulinemia who received IVIG, five patients (8.9%) had COVID-19 infection during the study period. In patients with hypogammaglobulinemia who did not receive IVIG, COVID-19 infection was developed in 8 of 33 (24.2%). In patients without hypogammaglobulinemia, COVID-19 infection was developed in 9 of 36 patients (22.2%) (**Supplementary Figure S1**). Patients receiving IVIG infusion demonstrated a significantly lower incidence of COVID-19 infection compared to those who did not receive IVIG (8.9% vs. 24.6%; p = 0.040) (**Figure 2**). The clinical course of patients infected with COVID-19 is summarized in **Supplementary Figure S2** and **Supplementary Table S3**. In the univariate analysis, age, ECOG performance status, Ann Arbor stage, IPI at diagnosis, hypogammaglobulinemia at any time during immunochemotherapy, and IVIG administration were associated with COVID-19 infection (**Supplementary Table S4**). However, in the multivariate analysis, only age > 80 years has a significantly increased risk of acquiring COVID-19 infection (hazard ratio [HR] 1.67, p = 0.041). Although IVIG administration demonstrated a protective tendency against COVID-19 infection, statistical significance was not reached (HR 0.40, p = 0.082) (**Figure 3**).

### 3.4 Newly developed hypogammaglobulinemia during reduced intensity R-CHOP therapy

Among patients with hypogammaglobulinemia who received IVIG, 19 of 47 patients (40.5%) developed additional immunoglobulin deficiencies during chemotherapy, primarily IgG (n = 10) and IgM (n = 10). In patients with hypogammaglobulinemia who did not receive IVIG, 18 out of 25 patients (72.0%) showed one or more new hypogammaglobulinemia, and IgG (n = 13), and IgM (n = 8) were most commonly identified. In patients who did not show hypogammaglobulinemia at diagnosis, 19 of 31 patients (61.3%) newly developed one or more classes of hypogammaglobulinemia during immunochemotherapy, and decreased IgG (n = 13), IgM (n = 10), IgD (n = 9), and IgA (n = 3) were identified.

### 3.5 Severe infections during reduced intensity R-CHOP therapy

Severe infection rates during reduced intensity R-CHOP therapy were considerably lower in patients receiving IVIG (p = 0.031). In the IVIG group, severe infections occurred in three patients (5.4%), while in the non-IVIG hypogammaglobulinemia group, eight patients (25.0%) experienced severe infections, and in patients without hypogammaglobulinemia, five patients (13.9%) had severe infections.

## 4 Discussion

In this study, the incidence of COVID-19 infection was relatively higher in patients who did not receive IVIG during reduced intensity R-CHOP therapy than in patients with IVIG. IVIG administration was generally well-tolerated, with majority of the adverse events being mild and manageable. Notably, only one patient discontinued IVIG infusion due to NSTEMI.

While numerous studies have investigated hypogammaglobulinemia in CLL, limited research has investigated its clinical significance in patients with DLBCL. Hypogammaglobulinemia, associated with compromised T- and nonclonal CD5- B cell functions, significantly impacts infection frequency and survival rates in patients with CLL (28, 29). Studies in DLBCL, such as those of Singh et al. and Pan et al., have reported lower rates of hypogammaglobulinemia (22.1% and 19%, respectively) and highlighted its association with unfavorable survival and increased infection risks (13, 15). Our study noted a higher prevalence of hypogammaglobulinemia (71.2%) at diagnosis, which could be attributed to the advanced median age of our targeted patient group (77 years) compared to previous studies (62–65 years). Furthermore, the inclusion of IgD deficiency, documented in approximately 50% of cases, might have contributed to a higher prevalence. Hypogammaglobulinemia incidence and subsequent infection rates appeared to increase with B-cell depletion induced by rituximab-containing chemoimmunotherapy (30, 31). Importantly, more than half of the patients, irrespective of their immunoglobulin levels at diagnosis, developed additional immunoglobulin deficiencies, mainly IgG and IgM, during reduced intensity R-CHOP therapy.

During chemotherapy, COVID-19 infection negatively impacted DLBCL treatment outcomes, often leading to treatment postponement or discontinuation. While our study rarely indicated the normalization of serum immunoglobulin levels with 400 mg/kg IVIG replacement during chemotherapy, multivariate analysis indicated a potential preventive role against COVID-19 infection. Previous randomized clinical trials in CLL also supported the efficacy of prophylactic IVIG in remarkably reducing infection rates compared to placebo (32, 33).

The introduction of SARS-CoV-2 vaccines was ongoing during patient enrollment. Owing to safety and efficacy concerns, elderly patients initially exhibited hesitancy toward vaccination, resulting in incomplete vaccination. Consequently, this study could not confirm the preventive effect of SARS-CoV-2 vaccination in patients with DLBCL or assess the advantages and disadvantages of IVIG administration alongside vaccination. While our data do not conclusively establish the clinical benefit of IVIG, this study underscores the promising potential of IVIG as prophylaxis in elderly patients with DLBCL. However, the results of the present data should be interpreted cautiously due to certain limitations. First, the current study was not randomized. There may be a risk of bias. Second, this study could not address data analysis of anti-SARS-CoV-2 titers before and after IVIG replacement. Third, sample size and number of events were relatively small for between-group comparisons. Plus, follow-up duration was short. Lastly, this study was conducted on elderly patients, and the number of cases in which the study was discontinued due to various factors was greater than estimated. Given the susceptibility of patients with malignant lymphoma receiving B cell–depleting therapy to COVID-19 and their higher mortality rates, a comprehensive randomized controlled study with appropriate IVIG dosing and administration schedules is warranted to further define IVIG’s role in preventing COVID-19 infections.

## Data availability statement

The original contributions presented in the study are included in the article/**Supplementary Material**. Further inquiries can be directed to the corresponding author.

## Ethics statement

The studies involving humans were approved by The Institutional Review Board of Kyungpook National University Hospital (KNUH 2020-03-026). The studies were conducted in accordance with the local legislation and institutional requirements. Written informed consent for participation in this study was provided by the participants’ legal guardians/next of kin.

## Author contributions

DB: Conceptualization, Data curation, Formal analysis, Investigation, Methodology, Project administration, Resources, Supervision, Validation, Visualization, Writing – original draft, Writing – review & editing. G-YS: Data curation, Investigation, Writing – original draft, Writing – review & editing. HL: Conceptualization, Investigation, Writing – original draft, Writing – review & editing. YD: Conceptualization, Investigation, Writing – original draft, Writing – review & editing. JL: Conceptualization, Investigation, Writing – original draft, Writing – review & editing. H-YY: Conceptualization, Investigation, Writing – original draft, Writing – review & editing. JM: Investigation, Methodology, Writing – original draft, Writing – review & editing. D-HY: Conceptualization, Data curation, Formal analysis, Investigation, Methodology, Project administration, Resources, Supervision, Validation, Visualization, Writing – original draft, Writing – review & editing.
