# Supplementary material for: Clinical efficacy of prophylactic intravenous immunoglobulin for elderly DLBCL patients with hypogammaglobulinemia in the COVID-19 pandemic era
Source: Front Oncol. 2024 Apr 23;14:1380492. doi: 10.3389/fonc.2024.1380492 (PMC11075150; doi:10.3389/fonc.2024.1380492)

Supplementary Material

# Supplementary Table

**Supplemental Table 1.** Treatment schedule of the patients with R-mini-CHOP plus intravenous immunoglobulin.

| **Cycle 1** | **Dose** | **D1** | **D2** | **D3** | **D4** | **D5** | **D11-14** |
| --- | --- | --- | --- | --- | --- | --- | --- |
| Rituximab | 375 mg/m^2^ | O |  |  |  |  |  |
| Cyclophosphamide | 600 mg/m^2^ | O |  |  |  |  |  |
| Doxorubicin | 30 mg/m^2^ | O |  |  |  |  |  |
| Vincristine | 1.0 mg/m^2^ | O |  |  |  |  |  |
| Prednisolone | 40 mg/m^2^ | O | O | O | O | O |  |
| Pegylated G-CSF | 6 mg/0.6 ml |  | O |  |  |  |  |
| IVIG | 400 mg/kg |  |  | O |  |  | O |
|  | | | | | | | |
| **Cycle 2-6** | **Dose** | **D1** | **D2** | **D3** | **D4** | **D5** | **D11-14** |
| Rituximab | 375 mg/m^2^ | O |  |  |  |  |  |
| Cyclophosphamide | 600 mg/m^2^ | O |  |  |  |  |  |
| Doxorubicin | 30 mg/m^2^ | O |  |  |  |  |  |
| Vincristine | 1.0 mg/m^2^ | O |  |  |  |  |  |
| Prednisolone | 40 mg/m^2^ | O | O | O | O | O |  |
| Pegylated G-CSF | 6 mg/0.6 ml |  | O |  |  |  |  |
| IVIG | 400 mg/kg |  |  |  |  |  | O |

Abbreviations: G-CSF, granulocyte-colony stimulating factor; IVIG, intravenous immunoglobulin

**Supplemental Table 2.** Reported events of adverse reactions during administration of intravenous immunoglobulin (n =56)

| **Toxicities** | **Grade 1-2, no. of cases** | **Grade 3-4, no. of cases** |
| --- | --- | --- |
| Chest pain | 0 | 1 |
| Chills | 7 | 0 |
| Vomiting | 4 | 0 |
| Headache | 4 | 0 |
| Tachycardia | 4 | 0 |
| Dyspnea | 3 | 0 |
| Nausea | 3 | 0 |
| Fatigue | 3 | 0 |
| Chest discomfort | 2 | 0 |
| Dizziness | 2 | 0 |
| Myalgia | 2 | 0 |
| Fever | 2 | 0 |
| Skin rash | 1 | 0 |
| Pain | 1 | 0 |
| Cough | 0 | 0 |
| Abdominal pain | 0 | 0 |
| Edema | 0 | 0 |
| Diarrhea | 0 | 0 |
| Dermatitis | 0 | 0 |
| Hypertension | 0 | 0 |
| Hypotension | 0 | 0 |

**Supplemental Table 3.** Clinical course of patients infected with COVID-19

| No. | Age | Sex | Stage | IPI | Hypogammaglobulinemia at diagnosis | IVIG | Newly developed hypogammaglobulinemia during chemotherapy | Completion of scheduled chemotherapy | Response assessment |
| --- | --- | --- | --- | --- | --- | --- | --- | --- | --- |
| 1 | 82 | F | 4 | 5 | IgD | administered | IgG, IgA | Delayed but completed | PR |
| 2 | 83 | F | 3 | 3 | IgG, IgD | administered | none | Discontinued after COVID-19 | - |
| 3 | 70 | F | 1 | 2 | IgM | administered | IgG | Completed | CR |
| 4 | 74 | M | 2 | 2 | IgM | administered | IgM | Completed | CR |
| 5 | 74 | M | 2 | 2 | IgG, IgM | administered | none | Discontinued after COVID-19 | PR |
| 6 | 71 | M | 4 | 5 | IgM, IgD | none | none | Delayed but completed | PD |
| 7 | 83 | F | 4 | 5 | IgM | none | none | Delayed but completed | CR |
| 8 | 70 | F | 4 | 4 | IgD | none | none | Completed | CR |
| 9 | 70 | M | 3 | 4 | IgA, IgD | none | IgG | Discontinued after COVID-19 | - |
| 10 | 79 | F | 3 | 4 | IgD | none | IgM, IgG | Discontinued after COVID-19 | - |
| 11 | 71 | M | 4 | 3 | IgM | none | IgG | Discontinued after COVID-19 | CR |
| 12 | 81 | M | 2 | 3 | IgM | none | IgG | Delayed but completed | CR |
| 13 | 70 | M | 2 | 3 | IgD | none | none | Completed | CR |
| 14 | 86 | M | 4 | 5 | none | none | IgG, IgM | Delayed but completed | PR |
| 15 | 70 | M | 4 | 5 | none | none | IgM | Delayed but completed | PD |
| 16 | 70 | F | 4 | 4 | none | none | IgG | Completed | CR |
| 17 | 79 | F | 3 | 4 | none | none | IgG, IgM | Discontinued after COVID-19 | - |
| 18 | 78 | F | 2 | 3 | none | none | none | Completed | CR |
| 19 | 78 | M | 3 | 3 | none | none | IgM | Delayed but completed | CR |
| 20 | 79 | F | 1 | 3 | none | none | IgM | Delayed but completed | CR |
| 21 | 74 | F | 3 | 3 | none | none | IgG, IgM | Discontinued after COVID-19 | - |
| 22 | 75 | M | 2 | 2 | none | none | IgG, IgM, IgD | Discontinued after COVID-19 | PD |

Abbreviations: COVID-19, coronavirus disease 2019; CR, complete response; IPI, international prognostic index; IVIG, intravenous immunoglobulin; PD, progressive disease; PR, partial response

**Supplemental Table 4.** Factors affecting COVID-19 infection during chemotherapy

|  | Univariate | | |  | Multivariate | | |
| --- | --- | --- | --- | --- | --- | --- | --- |
|  | HR | 95% CI | p-value |  | HR | 95% CI | p-value |
| Age at diagnosis, > 80 vs. ≤ 80 | 1.61 | 1.13-3.64 | 0.03 |  | 1.67 | 1.21-3.97 | 0.04 |
| Female vs. male | 0.72 | 0.29-1.77 | 0.47 |  |  |  |  |
| LDH, elevated vs. normal | 0.64 | 0.20-2.04 | 0.45 |  |  |  |  |
| ECOG, > 1 vs. ≤ 1 | 1.38 | 0.83-2.62 | 0.07 |  | 1.79 | 0.91-3.03 | 0.08 |
| Ann Arbor stage, > 2 vs. ≤ 2 | 1.91 | 0.53-6.96 | 0.09 |  | 1.42 | 0.62-7.18 | 0.16 |
| Extranodal involvement, yes vs. none | 1.82 | 1.29-2.24 | 0.71 |  |  |  |  |
| IPI, > 2 vs. ≤ 2 | 1.76 | 0.94-4.24 | 0.09 |  | 2.16 | 0.87-5.13 | 0.31 |
| Prophylactic antibiotics, yes vs. none | 0.37 | 0.21-8.87 | 0.87 |  |  |  |  |
| Prophylactic antiviral agent, yes vs. none | 1.11 | 0.69-5.44 | 0.94 |  |  |  |  |
| Prophylactic antifungal agent, yes vs. none | 0.78 | 0.34-1.95 | 0.51 |  |  |  |  |
| Prophylactic anti-PCP agent, yes vs. none | 2.37 | 0.72-4.86 | 0.20 |  |  |  |  |
| Hypogammaglobulinemia, yes vs. none | 1.99 | 0.95-3.85 | 0.08 |  | 2.55 | 0.75-4.27 | 0.19 |
| Administration of IVIG, yes vs. none | 0.37 | 0.11-1.30 | 0.07 |  | 0.40 | 0.15-1.11 | 0.08 |

Abbreviations: CI, confidence interval; COVID-19, coronavirus disease 2019; ECOG, eastern cooperative oncology group; HR, hazard ratio; IPI, international prognostic index; IVIG, intravenous immunoglobulin; LDH, lactate dehydrogenase; PCP, Pneumocystis Pneumonia

**Supplementary Figure 1.** Comparison of COVID-19 infection between three groups


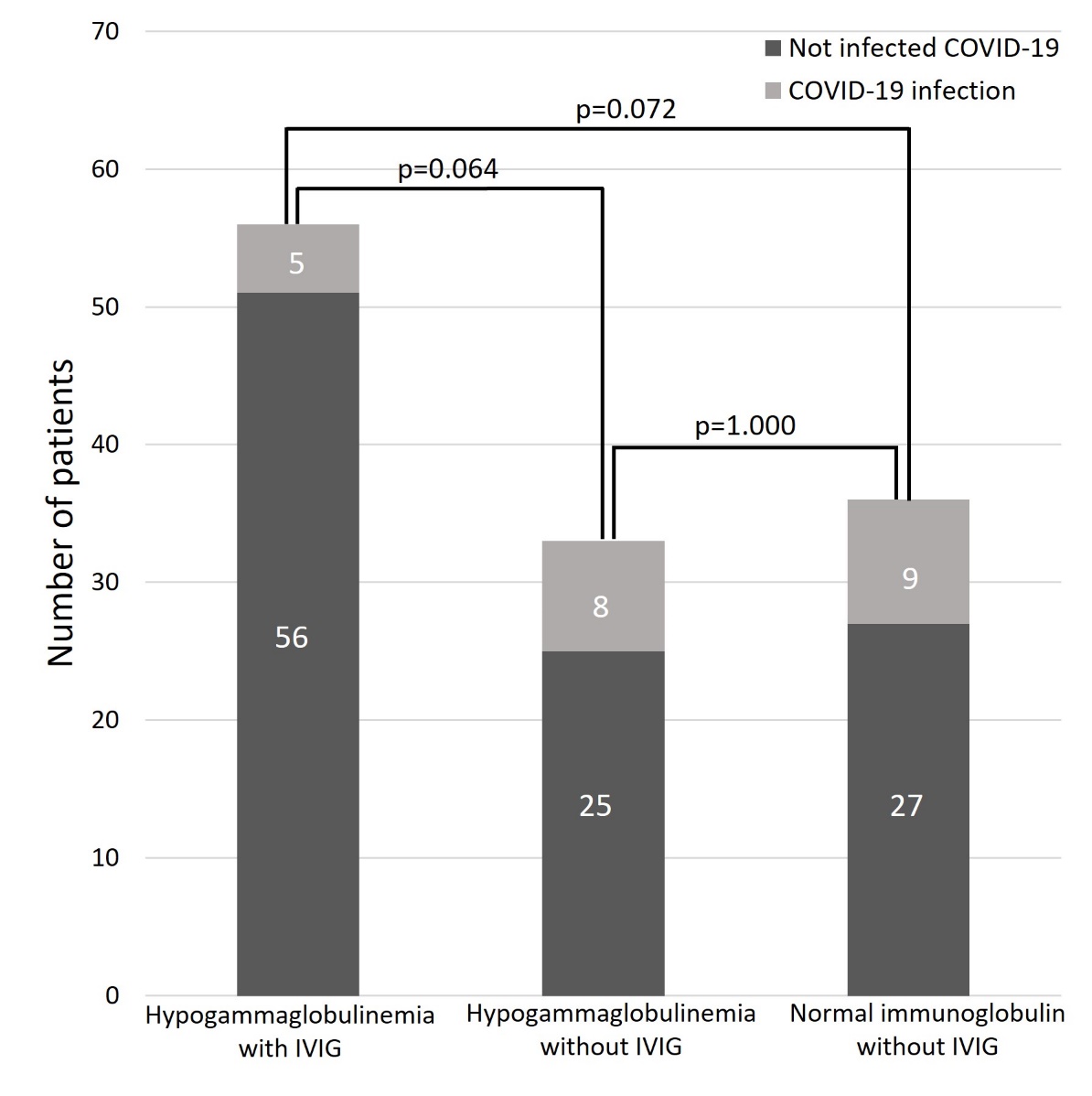


## Supplementary Figure 2. Timing of COVID-19 infection during chemotherapy
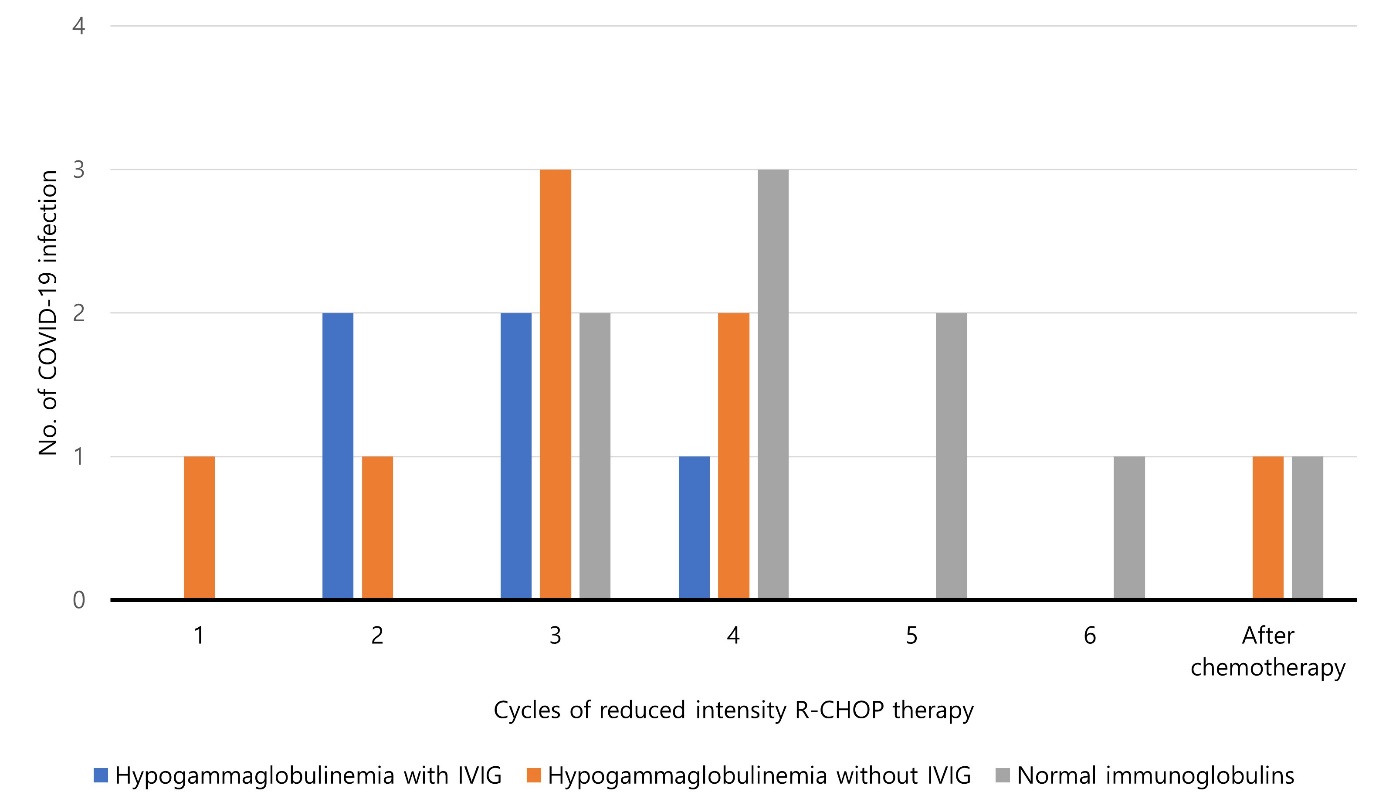

Supplement: Supplementary file 1 [file DataSheet_1.docx]
